# Study of Microbial Communities in the Soda Lake of Isabel Island: Identification of Polyhydroxybutyrate (PHB) Degrading Enzymes

**DOI:** 10.1111/1758-2229.70279

**Published:** 2026-02-17

**Authors:** Abigail Hernández‐Vázquez, Humberto Garcia‐Arellano, Rina María González‐Cervantes, Marcos López‐Pérez, Luis Mario Hernández Soto, José Abraham Canales Meza, José Félix Aguirre‐Garrido

**Affiliations:** ^1^ Doctorado en Ciencias Biológicas y de La Salud Universidad Autónoma Metropolitana Ciudad de México México; ^2^ Departamento de Ciencias Ambientales Universidad Autónoma Metropolitana‐Lerma Lerma Estado de México México; ^3^ Especialidad en Ciencias Naturales e Ingeniería Universidad Autónoma Metropolitana‐Cuajimalpa Ciudad de México México

**Keywords:** extremophiles, metagenomics, PHB depolymerases, soda lake, *Vreelandella*

## Abstract

Crater Lake (Isabel Island, Mexico) is a meromictic, stratified, haloalkaline system. To identify and characterise PHB depolymerases across the vertical physicochemical gradients of the lake, we analysed seven metagenomes from the water column (0–23 m), one sediment metagenome, and the genomes of two organisms (HB105m and VN105m) isolated from 5 m. Taxonomic profiles revealed vertical stratification: Actinobacteriota and Cyanobacteriota dominated surface waters, while Pseudomonadota, Bacillota, and Bacteroidota prevailed in deeper layers and sediments. Alpha‐diversity indices peaked at 5 and 20 m and declined at 23 m. We identified 16 putative PHB depolymerases spanning a broader phylogenetic range than previously documented for haloalkaline ecosystems. These included homologues affiliated with *Vreelandella*, *Thiomicrorhabdus*, Chloroflexota, Candidatus Cloacimonadota, and Desulfobacterales. The structural variation observed in lipase‐box motifs and signal peptides suggests functional differentiation linked to redox and oxygen gradients across depths. Phylogenetic analysis of predicted and reference enzymes showed depth‐specific clustering, with extracellular depolymerases predominant in oxic layers and intracellular forms more common in microoxic–anoxic zones. Overall, our results expand the known diversity of PHB‐degrading lineages in extreme environments and highlight several candidate enzymes with potential biotechnological relevance for future experimental characterisation.

## Graphical abstract

The crater lake on Isabel Island is a moderately saline soda lake that hosts microorganisms with potential biotechnological applications. We characterised the microbial communities using seven metagenomes and two genomes to identify polyhydroxybutyrate depolymerases. A total of 14 PHB depolymerases were identified in the metagenomes and two in the genomes.

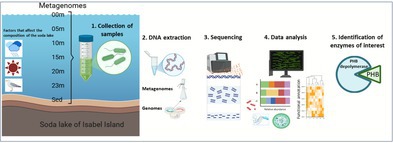

## 1 Introduction

### 1.1 Haloalkaline Environments and Biotechnological Potential

Haloalkaline habitats, characterised by high salinity and elevated pH, support diverse microbial communities adapted to extreme physicochemical conditions. These environments such as soda lakes, coastal evaporation zones, and saline deserts are recognised as reservoirs of novel enzymes with significant biotechnological potential (Sorokin et al. 2014; Muyzer et al. 2011). Among these enzymes are those involved in the metabolism of polyhydroxyalkanoates (PHAs), a family of biodegradable polyesters produced by extremophiles. Polyhydroxybutyrate (PHB), the most common PHA, has garnered particular interest due to its role in bioplastic production and its widespread occurrence in natural microbial communities (Chee et al. 2010).

### 1.2 Physiology and Ecological Roles of PHAs

PHAs serve multiple physiological functions in microorganisms: they act as intracellular carbon and energy reserves under nutrient‐limited conditions and provide protection against diverse environmental stresses, including osmotic imbalance, extreme pH, thermal fluctuations, and radiation (Obruca et al. 2020). These polymers are stored as cytoplasmic granules, surrounded by a phospholipid monolayer that contains polymerases and depolymerases that coordinate their synthesis and mobilisation (Jendrossek et al. 1996). PHAs are commonly classified by monomer length into short‐chain (3–5 carbons), medium‐chain (6–15 carbons), and long‐chain variants, with PHB representing the canonical short‐chain member. While PHB synthesis and regulation in haloalkaline microorganisms have been extensively documented (Rodríguez et al. 2024), much less is known about PHB degradation pathways in natural haloalkaline systems.

### 1.3 PHB Depolymerases: Types, Structure, and Knowledge Gaps

PHB degradation is mediated by PHB depolymerases, members of the α/β‐hydrolase fold family that exhibit high substrate specificity (Knoll et al. 2009). These enzymes occur in two main forms: intracellular depolymerases that degrade native amorphous PHAs within the cell, and extracellular depolymerases that hydrolyze semicrystalline or exogenous PHB commonly released after microbial cell lysis (Jendrossek and Handrick 2002). The catalytic domain of PHB depolymerases contains a conserved Ser‐Asp‐His triad and the characteristic lipase box (Gly‐X‐Ser‐X‐Gly), motifs typical of serine hydrolases (Jendrossek et al. 1996; Jaeger et al. 1994). Despite their ecological relevance, most existing knowledge of PHB depolymerases comes from a limited number of cultivable taxa, particularly *Halomonas*, leaving their broader diversity in natural soda lake environments poorly understood.

Recently, the taxonomic framework of Halomonadaceae underwent a major revision that has direct implications for interpreting PHB depolymerase diversity in extreme environments. In a comprehensive phylogenomic analysis, De La Haba et al. (2023) proposed the reclassification of *Halomonas aquamarina* and several phylogenetically related species into the newly established genus *Vreelandella*, forming a strongly supported monophyletic clade. Members of *Vreelandella* exhibit genomic signatures linked to osmoadaptation, oxidative stress tolerance, and the degradation of organic substrates characteristic of detritus‐rich hypersaline environments (Zhang et al. 2022; Wang et al. 2022); moreover, some strains of *Vreelandella* have been reported to produce PHA (Darden et al. 2025; Mitra et al. 2020). In this context, the PHB depolymerases associated with *Vreelandella* identified in this study gain particular relevance, as they may reflect the metabolic plasticity of this group. Although functional validation is still lacking, it is reasonable to hypothesise that *Vreelandella* contributes to polymer remineralization and carbon cycling in extreme saline and alkaline environments such as Crater Lake.

### 1.4 Crater Lake as a Model Haloalkaline Ecosystem

Crater Lake (Laguna Fragatas), located on Isla Isabel in the Mexican Pacific, is a moderately saline, alkaline, and meromictic soda lake whose chemistry is shaped by evaporation, precipitation, and nutrient inputs derived from seabird excreta (Aguirre‐Garrido et al. 2016; Hernández‐Soto et al. 2023). Previous research provided an initial characterisation of the surface microbial community at 1 m depth, offering a baseline description of dominant phototrophic and heterotrophic groups (Aguirre‐Garrido et al. 2016). However, that study did not explore the lake's vertical microbial structure, nor the metabolic capacities of subsurface populations, leaving fundamental questions unanswered regarding carbon cycling and polymer degradation along the lake's steep physicochemical gradients.

### 1.5 Ecological Relevance of PHAs and Depolymerases in Extreme Environments

In haloalkaline ecosystems, PHA depolymerases play an important role in microbial adaptation and carbon cycling. Beyond mobilising intracellular reserves, PHA degradation releases metabolites such as 3‐hydroxybutyrate that function as osmoprotectants and chemical chaperones under high salinity and alkaline stress (Obruca et al. 2020; Koller et al. 2018). Extracellular depolymerases further contribute to community‐level carbon turnover by hydrolyzing polymeric substrates in particulate matter or detrital biomass, generating diffusible compounds that support cross‐feeding interactions (D'Souza et al. 2023). Because soda lakes exhibit strong gradients in salinity, pH, oxygen, and redox chemistry, different depolymerase strategies may confer selective advantages across microhabitats. Yet, despite their potential importance, no study has examined the diversity or functional distribution of PHB depolymerases across the full water column of a haloalkaline lake.

### 1.6 Knowledge Gap, Hypothesis, and Study Objectives

To address this gap, we propose that Crater Lake harbours previously unrecognised lineages of PHB depolymerases whose phylogenetic affiliations, catalytic motifs, and depth‐dependent distribution reflect functional adaptations to the lake's steep physicochemical gradients. To test this hypothesis, we analysed metagenomes collected across multiple depths (0–23 m) and the sediment, as well as genomes from two isolates obtained at 5 m. Our goal was to identify PHB depolymerase genes, characterise their phylogenetic relationships, and examine their structural features including lipase boxes and signal peptides. By integrating depth‐resolved genomic and metagenomic data, this study provides the first comprehensive assessment of PHB depolymerase diversity in a natural haloalkaline ecosystem, revealing novel enzymatic lineages with ecological significance and potential biotechnological applications.

## 2 Material and Methods

### 2.1 Sample Collection

One litre of water or sediment was collected for each sample in a pre‐sterilised PET bottle. Replicate samples could not be obtained because Crater Lake is located within a Mexican National Park designated as a Protected Natural Area (ANP), where access is restricted and boat space for sampling is limited. Water for genomic and metagenomic extraction was collected using a Van Dorn bottle, while the sediment sample was obtained with an Ekman dredge. Samples were taken at 0, 5, 10, 15, 20, 23 m, and from sediment (Sed) in Fragatas Lagoon, on Isabel Island, Nayarit, Mexico (21.846621 N, 105.883377 W).

Strains HB105m and VN105m were selected to complement the metagenomic analysis, providing high‐quality genomes for the detection of depolymerases. These isolates were obtained through serial dilutions in a halophilic medium (HB) containing: 100 g/L NaCl, 25 g/L MgSO_4_·7H_2_O, 10 g/L casamino acids, 10 g/L yeast extract, 5 g/L proteose peptone, 3 g/L trisodium citrate, 2 g/L KCl (pH 8.5), and in Van Niel (VN) medium containing: 100 g/L NaCl, 20 g/L agar, 10 g/L yeast extract, 1 g/L K_2_HPO_4_, 0.5 g/L MgSO_4_ (pH 8.5). After several passages, strains were identified by Sanger sequencing of the 16S rRNA gene. Genomes from HB105m and VN105m, as well as seven metagenomes from the water column and sediment, were analysed for the presence of PHB depolymerases.

### 2.2 Genome and Metagenome Sequencing and Bioinformatic Analysis

Genomic and metagenomic DNA sequencing was conducted at the CGEB‐Integrated Microbiome Resource sequencing unit at Dalhousie University, Canada. Libraries were prepared using the Nextera Flex kit and sequenced on the Illumina MiSeq platform (150 × 150 bp paired‐end) (Comeau et al. 2017).

Raw reads were assessed with FastQC (2011). Reads with Phred scores < 20 or lengths < 146 bp were removed using Trimmomatic (Bolger et al. 2014). The assembly of the metagenomes and genomes was performed with Unicycler (Wick et al. 2017), and contigs < 200 bp were discarded. The quality of the metagenomic and genomic assemblies was evaluated using QUAST (Gurevich et al. 2013) and standard assembly metrics (N50, L50, total assembled bp, No. Contigs, largest contig, average coverage depth and coverage ≥ 10×).

**FIGURE 1:**
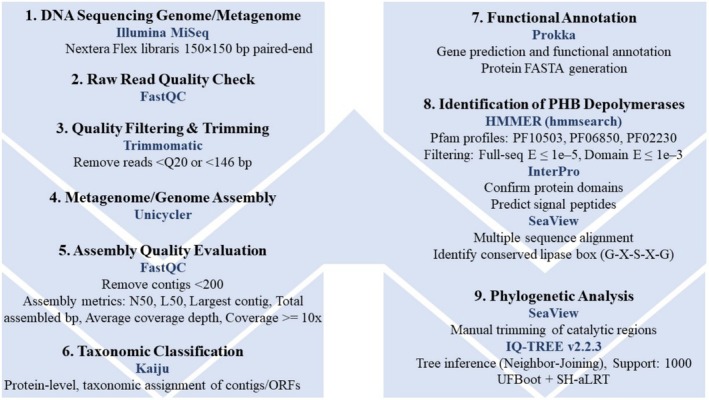
Bioinformatic workflow used for the processing of genome and metagenome data. The pipeline includes raw‐read quality assessment (FastQC), quality filtering (Trimmomatic), genome and metagenome assembly (Unicycler), assembly quality evaluation, taxonomic assignment (Kaiju), functional annotation (Prokka), identification of PHB depolymerases (HMMER, InterPro, SeaView), and phylogenetic inference (SeaView and IQ‐TREE).

Clean metagenome assemblies were used as input for Kaiju (Menzel et al. 2016) for taxonomic classification and for Prokka (Seemann 2014) for functional annotation. Genomes were also annotated using Prokka. Figure 1 presents a flowchart summarising the bioinformatic workflow followed in this study, encompassing the steps described in Sections 2.2, 2.4.

### 2.3 Identification of PHB Depolymerases

Annotated proteins for each metagenome and genome were scanned with HMMER (Potter et al. 2018) using Pfam HMM profiles PF10503 (PHB‐type esterase depolymerases), PF06850 (C‐terminal substrate‐binding domain of bacterial PHB depolymerases), and PF02230 (phospholipases and carboxylesterases) (Knoll et al. 2009). Hits were considered significant when full‐sequence *E*‐value ≤ 1e‐5 and/or domain *E*‐value ≤ 1e‐3. Candidate sequences were manually inspected with InterPro (Hunter et al. 2009) to confirm domain and identify signal peptides. Multiple‐sequence alignment was generated using SeaView (Gouy et al. 2010) to verify the presence of the conserved lipase box (G‐X‐S‐X‐G).

### 2.4 Phylogenetic Tree of PHB Genes Identified in Genomes and Metagenomes

A custom‐built database was constructed containing previously reported bacterial depolymerases (NCBI) and all predicted PHB depolymerase sequences identified in this study. Amino acid sequences were aligned and manually trimmed in SeaView (Gouy et al. 2010) to retain conserved catalytic domains. Phylogenetic inference was conducted using IQ‐TREE v2.2.3 (Nguyen et al. 2015) under the Neighbour‐Joining method.

ModelFinder selected WAG + F + R4 as the best‐fit amino acid substitution model according to the Bayesian Information Criterion (BIC). Node support was assessed using 1000 ultrafast‐bootstrap replicates (UFBoot) and SH‐aLRT tests, with support values ≥ 95% considered statistically reliable.

## 3 Results

### 3.1 Metagenome and Genome Assembly

For each metagenome and each genome, we reported the total number of paired reads generated; the reads surviving after filtering (Phred > 20, length ≥ 146 bp); the total assembly length (bp); the number of contigs; the length of the longest contig; the N50 and L50 values; the mean coverage; and the proportion of bases with ≥ 10× coverage (Table 1 for metagenomes; Table 2 for genomes).

**TABLE 1:** Quality parameters and assembly metrics of the metagenomes obtained from different depths in Isabel Island Crater Lake.

Metagenome	Raw reads (pairs)	Reads post‐QC	Total assembled bp	No. Contigs	Largest contig	N50	L50	Avg coverage depth	Coverage ≥ 10× (%)
0 m	5,544,218	5,470,540	15,993,352	2294	355,545	17,051	191	22	89
5 m	4,116,238	4,094,504	8,981,540	708	209,238	25,304	96	24	85.43
10 m	4,082,896	4,075,510	10,791,247	3047	53,271	5139	468	12	62.96
15 m	5,113,476	5,105,832	14,023,698	2710	73,349	10,476	362	12	65.87
20 m	2,401,064	2,397,698	3,954,044	1218	26,128	4569	252	10	50.19
23 m	4,468,198	4,438,640	6,542,466	3103	23,256	2272	884	7	21
Sed	5,087,224	5,075,998	7,739,322	2408	109,617	4187	371	14	69.4

**TABLE 2:** Quality metrics of the genome assemblies for the bacterial isolates HB105m and VN105m recovered at 5 m depth from Isabel Island Crater Lake.

Genome	Raw reads (pairs)	Reads post‐QC	Total assembled bp	No. Contigs	Largest contig	N50	L50	Avg coverage depth	Coverage ≥ 10× (%)
HB105m	1,881,643	1,881,643	3,915,794	46	611,157	289,986	5	106	100
VN105m	1,704,558	1,704,558	3,837,928	39	836,516	337,596	4	105	100

Across all metagenomes, 90%–99% of raw reads passed quality filtering, indicating high sequencing accuracy and minimal loss of usable data (Table 1). Assemblies from Crater Lake exhibited depth‐dependent variation in performance. Surface metagenomes (0 and 5 m) showed the best assembly metrics, with mean coverages of 22× and 24×, respectively, more than 85% of bases covered at ≥ 10×, the longest contigs (355 and 209 kb), and the highest N50 values (17–25 kb). In contrast, mid‐ and deep‐water metagenomes (10–23 m) displayed lower sequencing depth (7–12×) and a reduced fraction of bases at ≥ 10× coverage (21%–66%). The sediment metagenome showed intermediate assembly quality, with 14× mean coverage and 69% of bases covered at ≥ 10×.

For the genomes of the bacterial isolates HB105m and VN105m, obtained from the 5 m water sample of Isla Isabel Crater Lake, the assemblies showed high completeness and continuity (Table 2). After quality filtering, 100% of reads were retained for both genomes. The assemblies resulted in sizes of 3.9 Mb (HB105m) and 3.8 Mb (VN105m), with 46 and 39 contigs, respectively. The longest contigs measured 611 kb (HB105m) and 836 kb (VN105m), and the N50 values were 289 and 337 kb, respectively. Both genomes also exhibited > 100× mean coverage and 100% of bases with ≥ 10× coverage.

### 3.2 Relative Abundance of Bacterial Populations in Metagenomes

**FIGURE 2:**
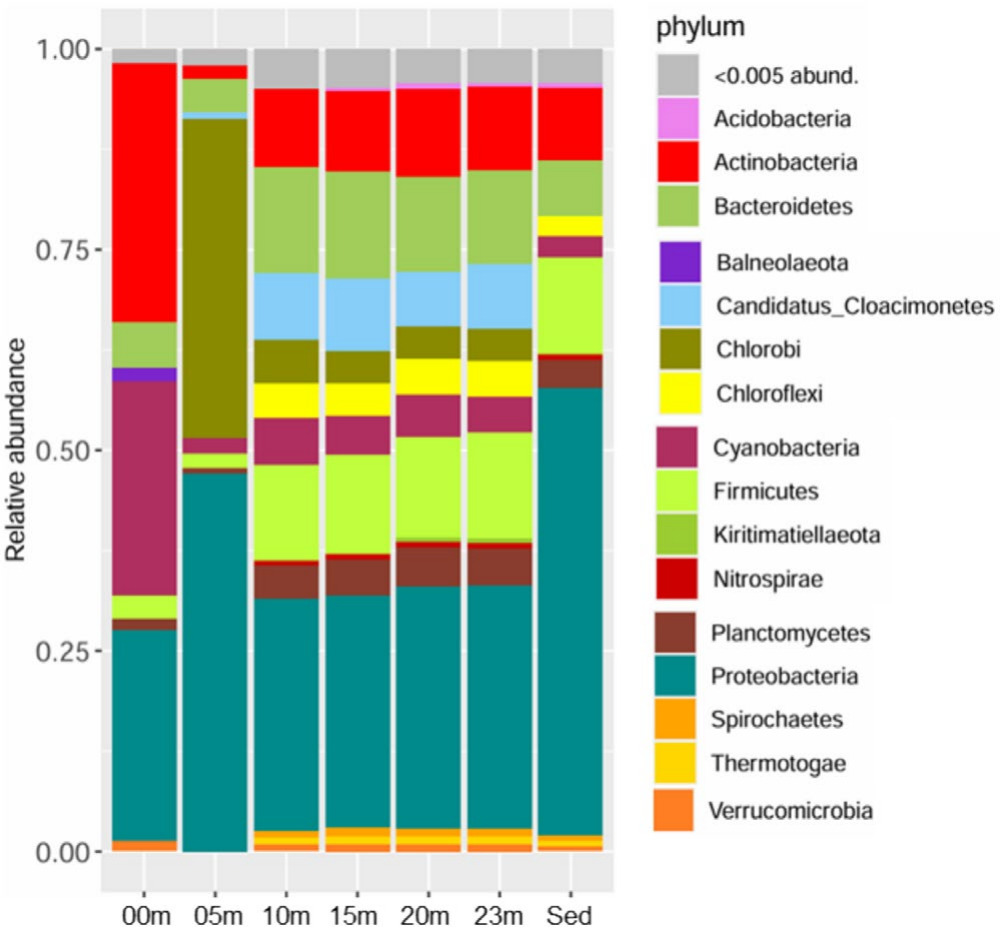
Relative abundance of populations filtered by phylum in the metagenomes of the soda lake on Isabel Island.

Figure 2 shows the relative abundance of bacterial populations at the phylum level across all metagenomes, revealing a clear vertical stratification. The surface metagenome (0 m) is dominated by Actinobacteriota (0.324), Cyanobacteriota (0.266), and Pseudomonadota (0.265), reflecting the photic, oxygenated conditions. At 5 m, the community shifts sharply, with Pseudomonadota (0.470), Chlorobiota (0.398), and Bacteroidota (0.0422) emerging as the most abundant phyla. In the sediment sample, Pseudomonadota (0.558), Bacillota (0.122), and Actinobacteriota (0.090) are the dominant phyla, indicating a community structure distinct from both surface and mid‐depth waters.

In contrast, the communities at intermediate depths (10, 15, 20, and 23 m) exhibit less variation in dominant phyla and share a similar structure. Across these depths, Pseudomonadota (average: 0.1261 ± 0.00831), Bacillota (average: 0.1263 ± 0.00534), and Bacteroidota (average: 0.2967 ± 0.00788) consistently predominate. Despite this stability in dominant groups, these mid‐depth metagenomes display a higher number of detected phyla (13–15 per sample) compared to 0 and 5 m (both with 8 phyla) and to the sediment (12 phyla), indicating greater taxonomic richness at depth.

Actinobacteriota show a pronounced decline from the surface (0 m: 0.3241) to deeper waters (0.017–0.110), whereas Bacillota exhibit the opposite pattern, increasing steadily with depth (0.12–0.13 at 10–23 m and 0.122 in sediments) relative to surface samples (0.029 at 0 m and 0.019 at 5 m). This shift suggests a transition from phototrophic and aerobic lineages near the surface to heterotrophic, fermentative, or anaerobic groups in deeper layers and sediments.

### 3.3 Diversity Indices Across Depth

The Shannon and inverse Simpson indices (Table 3) reinforce the stratification patterns observed in the relative abundances. The diversity indices exhibited a clearly stratified pattern throughout the water column of Crater Lake. Diversity increased at 5 and 20 m, where the highest Shannon (2.8) and inverse Simpson values (4.7 and 2.5, respectively) were recorded, indicating relatively more even communities in these intermediate layers. In contrast, diversity decreased markedly at 23 m (Shannon 2.2; Simpson 1.9), coinciding with the anoxic zone. The surface (0 m), the 10–15 m layers, and the sediment showed intermediate values (Shannon 2.6–2.7; inverse Simpson 2.3–2.8), suggesting diverse communities but with greater dominance of certain taxa.

**TABLE 3:** Shannon and Inverse Simpson diversity indices calculated for each metagenome depth.

Metagenome	Shannon diversity index (H′)	Inverse Simpson index (1/D)
0 m	2.6	2.8
5 m	2.8	4.7
10 m	2.6	2.3
15 m	2.7	2.4
20 m	2.8	2.5
23 m	2.2	1.9
Sed	2.7	2.4

### 3.4 PHB Depolymerases Identified in Metagenomes and Genomes

The phbZ gene was sought, which is involved in the degradation of polyhydroxyalkanoates (PHA), specifically in the degradation of polyhydroxybutyrate (PHB), the most common PHA in nature. Table 4 shows the PHB depolymerases identified in the Crater Lake metagenomes. A total of 14 PHB depolymerases were predicted in the metagenomes: five were present in the 05 m metagenome, four in the Sed metagenome, two in the 15 m metagenome, and one in the 10 and 20 m metagenomes, respectively. Two of the five depolymerases from the 05 m metagenome (05 m_PhbZV1, 05 m_PhbZV2) and one of the four depolymerases from the Sed metagenome (Sed_PhbZV4) were associated with the genus *Vreelandella*, with identities of 87.6%, 78.27%, and 81.052%, respectively. For the depolymerases identified in the metagenomes, the dominant phyla are Pseudomonadota, Chloroflexota, and Thermodesulfobacteriota. At the class level, eight depolymerases (00 m_PhbZW1, 05 m_PhbZP1, 05 m_PhbZT1, 05 m_PhbZT2, Sed_PhbZT3, 05 m_PhbZV1, 05 m_PhbZV2, Sed_PhbZV3) could be assigned to Gammaproteobacteria, and two to Desulfobacteria (15 m_PhbZDf1, 15 m_PhbZDt1).

**TABLE 4:** Depolymerases of PHB identified in the metagenomes of Crater Lake of Isabel Island.

Metagenome	Predicted PHB depolymerase^a^	Microorganism with which it was associated	Query cover %	Percent identity %	Signal peptide	Predicted PHB depolymerase type
0 m	00 m_PhbZW1	*Wenzhouxiangella* sp.	99	75.53	No	Intracellular
5 m	05 m_PhbZP1	*Pseudoalteromonas*	100	98.62	No	Intracellular
	05 m_PhbZT1	*Thiomicrorhabdus indica*	78	64	No	Intracellular
	05 m_PhbZT2	*Thiomicrorhabdus indica*	94	50	No	Intracellular
	05 m_PhbZV1	*Vreelandella aquamarina*	100	87.6	Yes	Extracellular
	05 m_PhbZV2	*Vreelandella aquamarina*	99	78.27	Yes	Extracellular
10 m	10 m_PhbZCa1	Phylum Chloroflexota	98	77.25	No	Intracellular
15 m	15 m_PhbZDf1	Order Desulfobacterales	77	66	No	Intracellular
	15 m_PhbZDt1	Order Desulfatiglandales	98	82.72	No	Intracellular
20 m	20 m_PhbZC1	Phylum Candidatus Cloacimonadota	100	92.2	Yes	Extracellular
Sed	Sed_PhbZCa2	Phylum Chloroflexota	100	71	Yes	Extracellular
	Sed_PhbZCi1	Phylum Chloroflexota	100	77.27	No	Intracellular
	Sed_PhbZT3	*Thiomicrorhabdus indica*	97	49.76	No	Intracellular
	Sed_PhbZV3	*Vreelandella aquamarina*	97	81.05	No	Intracellular

^a^ The names of the predicted PHB depolymerases follow the structure [depth]_PhbZ_[taxon abbreviation]. The first element indicates the depth of the metagenome in which the sequence was identified (e.g., 00 m = 0 m). The prefix PhbZ denotes that the sequence corresponds to a putative PHB depolymerase. The final letter or combination of letters represents the abbreviation of the microorganism with which the sequence showed the highest similarity. For example, in 00 m_PhbZW1, 00 m indicates the sampling depth, PhbZ refers to the putative function, and W corresponds to *Wenzhouxiangella*, the genus with which the sequence was associated.

In the case of the genomes, a PHB depolymerase was predicted for VN105m (VN105m_PhbZV4) and HB105m (HB105m_PhbZV5), which were associated in the Gammaproteobacteria class, specifically in the genus *Vreelandella*, with 78% and 78.55% identity, respectively (Table 5).

**TABLE 5:** PHB depolymerases identified in the genomes of the Crater Lake of Isabel Island.

Genome	Predicted PHB depolymerase^ª^	Microorganism with which it was associated	Query cover %	Percent identity %	Signal peptide	Predicted PHB depolymerase type
VN105m	VN105m_PhbZV4	*Vreelandella aquamarina*	99	78.55	Yes	Extracellular
HB105m	HB105m_PhbZV5	*Vreelandella aquamarina*	99	78.55	Yes	Extracellular

^a^ The predicted PHB depolymerases VN105m_PhbZV4 and HB105m_PhbZV5 follow the naming structure [genome ID]_PhbZ_[taxon abbreviation]. ‘VN105m’ and ‘HB105m’ correspond to the genome identifiers of isolates obtained from 5 m depth, PhbZ indicates a putative PHB depolymerase, and ‘*V*’ denotes their association with the genus *Vreelandella*.

**FIGURE 3:**
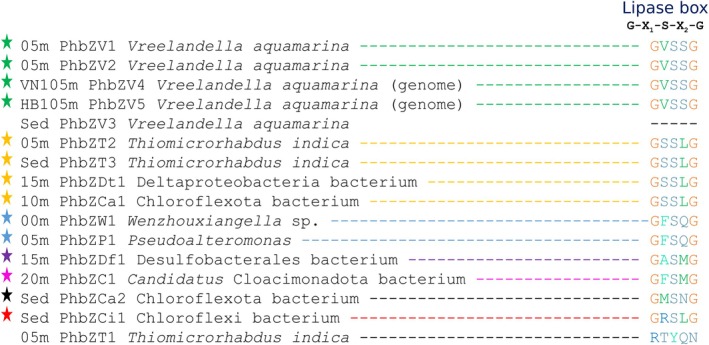
Identification of the lipase box in the PHB depolymerase sequences from metagenomes and genomes.

The sequences of PHB depolymerases from metagenomes and genomes were aligned to identify the lipase box. Figure 3 shows the identified lipase boxes in the sequences of PHB depolymerases from metagenomes and genomes, suggesting that these PHB depolymerases are potentially functional. The colours of the stars indicate different lipase boxes. In the metagenomes, the lipase box was identified in 12 of the 14 PHB depolymerases. In the two genomes, the lipase box was present in both PHB depolymerases. Seven types of lipase boxes were identified. Among them, the lipase box **GVSSG** was found in the depolymerases from metagenomes and genomes associated with the genus *Vreelandella* (05 m_PhbZV1, 05 m_PhbZV2, VN105m_PhbZV4, HB105m_PhbZV5). However, the depolymerase Sed_PhbZV3, also associated with the genus *Vreelandella*, did not present a lipase box. On the other hand, the lipase box **GSSLG** was present in the depolymerases 05 m_PhbZT2, Sed_PhbZV3, 15 m_PhbZDt1, and 10 m_PhbZCa1, associated with the genus *T. indica*, class Deltaproteobacteria, and phylum Chloroflexota. The lipase box **GFSQG** was identified in the depolymerases 00 m_PhbZW1 and 05 m_PhbZP1, associated with the genera *Wenzhouxiangella* and *Pseudoalteromonas*. The lipase box **GASMG** was found in the depolymerase 15 m_PhbZDf1, associated with the order Desulfobacterales. The lipase box **GFSMG** was present in the depolymerase 20 m_PhbZC1, associated with the phylum Candidatus Cloacimonadota. The lipase box **GMSNG** was present in the depolymerase Sed_PhbZCa2, associated with the phylum Chloroflexota, and the lipase box **GRSLG** in the depolymerase Sed_PhbZCi1, associated with the phylum Chloroflexota. Finally, in the depolymerase 05 m_PhbZT1, associated with the genus *T. indica*, the lipase box was also not identified. Additionally, in the PHB depolymerases 05 m_PhbZV1, 05 m_PhbZV2, 20 m_PhbZC1, Sed_PhbZCa2, VN105m_PhbZV4, and HB105m_PhbZV5, a characteristic signal peptide of extracellular PHB depolymerases was identified, suggesting that these depolymerases are extracellular.

### 3.5 Phylogeny of PHB Depolymerases

**FIGURE 4:**
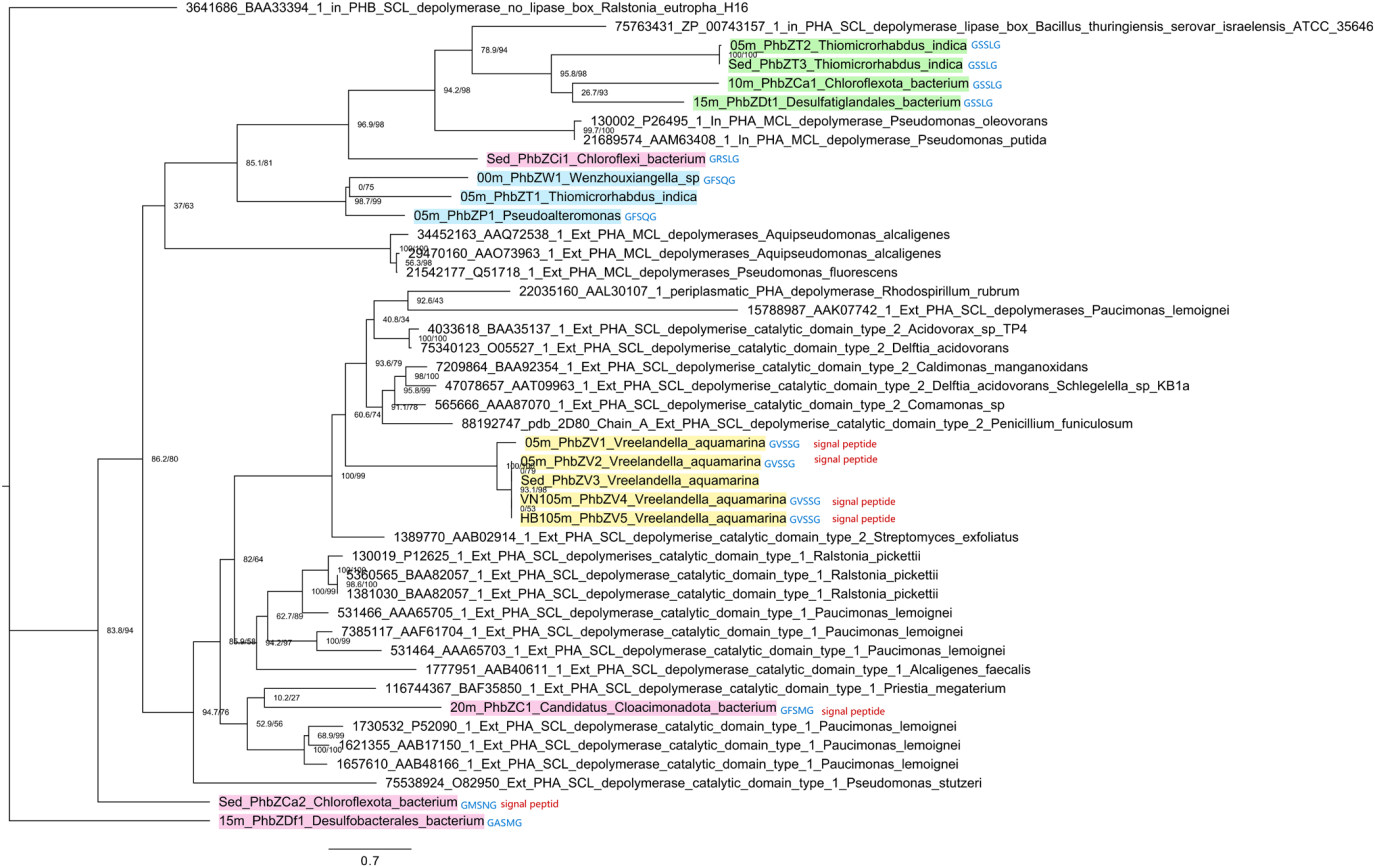
Unrooted phylogenetic tree, the outgroup of *Ralstonia eutropha* H16 is shown for reference only. Constructed using the maximum likelihood (ML) method with IQ‐TREE, with support values from 1000 bootstrap replicates and SH‐aLRT. The numerical values on the nodes correspond to the SH‐aLRT support (%) and ultrafast bootstrap support (%). The PHB depolymerases identified in this study are highlighted in green, pink, blue, and yellow.

The phylogenetic tree (Figure 4) obtained from the putative PHB depolymerases identified in metagenomes and genomes is unrooted. However, the extracellular depolymerase sequence from *Ralstonia eutropha* H16 (GenBank accession: BAA33394.1) was included as a reference outgroup and placed in the basal position solely for visual orientation. The numerical values on the nodes correspond to SH‐aLRT support (%) and ultrafast bootstrap (%) calculated with IQ‐TREE; overall, the main nodes showed support values above 80% and 95%, respectively.

The PHB depolymerases recovered in this study are highlighted in green, pink, blue, and yellow. The remaining, non‐highlighted sequences correspond to previously reported PHB depolymerases (Knoll et al. 2009), which are well characterised regarding their cellular localization (intracellular [in] vs. extracellular [Ext]), substrate preference (short‐chain‐length [SCL] vs. medium‐chain‐length [MCL]), and catalytic domain type (types 1 and 2). These well‐annotated reference sequences were used to guide the classification and interpretation of the environmental sequences recovered here. Based on this framework, the resulting tree reveals several well‐supported clades described below.

Depolymerase sequences affiliated with *V. aquamarina* (highlighted in yellow: 05 m_PhbZV1, 05 m_PhbZV2, Sed_PhbZV3, VN105m_PhbZV4, and HB105m_PhbZV5) formed a monophyletic clade with strong statistical support, clustering with extracellular short‐chain‐length (ext_PHA_SCL) depolymerases of type 1 and type 2 previously described in *Paucimonas lemoignei* , *Delftia acidovorans* , *Comamonas* sp., *Streptomyces exfoliatus* , and *Ralstonia pickettii*. Most sequences within this group (05 m_PhbZV1, 05 m_PhbZV2, VN105m_PhbZV4, and HB105m_PhbZV5) contained both a predicted signal peptide and the conserved GVSSG lipase box. In contrast, the Sed_PhbZV3 sequence, despite its proximity to this cluster, lacked both the signal peptide and the lipase box motif.

Sequences 05 m_PhbZT2 and Sed_PhbZT3 (affiliated with *T. indica*), together with 10 m_PhbZCa1 (a bacterium of phylum Chloroflexota) and 15 m_PhbZDt1 (a bacterium of order Desulfatiglandales), formed a separate well‐supported clade (SH‐aLRT = 95.8; ultrafast bootstrap = 98). This group branched as a sister lineage to intracellular short‐chain‐length (in_PHA_SCL) depolymerases from *Bacillus thuringiensis* . All sequences in this clade shared the GSSLG lipase box and lacked a signal peptide.

Another cluster included sequences 00 m_PhbZW1 (*Wenzhouxiangella* sp.), 05 m_PhbZT1 (*T. indica*), 05 m_PhbZP1 (*Pseudoalteromonas* sp.), and Sed_PhbZCi1 (a bacterium of phylum Chloroflexota), which grouped with intracellular medium‐chain‐length (in_PHA_MCL) depolymerases from *Pseudomonas putida* and *P. oleovorans* . Three of these sequences (00 m_PhbZW1, 05 m_PhbZP1, Sed_PhbZCi1) retained conserved lipase boxes (GFSQG, GFSQG, and GRSLG, respectively), and none encoded a signal peptide.

Finally, sequences 20 m_PhbZC1 (a bacterium of phylum Candidatus Cloacimonadota), Sed_PhbZCa2 (a bacterium of phylum Chloroflexota), and 15 m_PhbZDf1 (a bacterium of order Desulfobacterales) did not cluster with any established depolymerase groups. Nonetheless, all exhibited conserved lipase motifs (GFSMG, GMSNG, and GASMG, respectively); two sequences (20 m_PhbC1 and Sed_PhbZCa2) also contained predicted signal peptide.

## 4 Discussion

### 4.1 Relative Abundance of Bacterial Populations

The taxonomic patterns observed across the Crater Lake metagenomes are consistent with those reported for other haloalkaline systems and with the only previous study conducted in this lake. Aguirre‐Garrido et al. (2016) conducted the first microbial communities characterisation of Crater Lake of Isabel island and documented a marked differentiation between the water column and the sediments. In the water, the community was dominated by γ‐Proteobacteria, particularly Halomonadaceae, along with Cyanobacteriota and halophilic genera such as *Halomonas*, *Idiomarina*, and *Marinobacter*. These previously reported stratification patterns are consistent with the community structure observed in our metagenomes at different depths. Likewise, the predominance of Bacillota, Pseudomonadota, and Actinobacteriota in our sediment metagenome agrees with findings from Lake Lonar (Wani et al. 2006), and the alkaline Lake Hamatai (Liu et al. 2023), suggesting that these phyla consistently dominate the benthic communities of soda lakes.

Depth‐dependent taxonomic transitions comparable to those seen here have also been reported in meromictic soda systems such as Mono Lake, where Actinobacteriota dominate oxygenated surface waters while Bacillota prevail in deeper, chemically stratified layers (Humayoun et al. 2003). A similar pattern emerges in Crater Lake: a surface community enriched in Actinobacteriota shifts with depth toward Pseudomonadota, Bacillota, and Bacteroidota dominated communities. These transitions are consistent with the lake's pronounced physicochemical gradients, particularly salinity, oxygen availability, and energy sources, which are known to shape microbial structure in haloalkaline environments.

The consistent presence of Pseudomonadota across all depths is also ecologically coherent given their metabolic versatility and tolerance to hypersaline, nutrient‐limited conditions (Jendrossek and Handrick 2002; Shimao 2001; Park et al. 2021). Considering that only ~35 PHB depolymerases had been biochemically characterised by 2021, most from Pseudomonadota (Viljakainen and Hug 2021), their high relative abundance in Crater Lake suggests substantial potential for active PHA turnover. Gammaproteobacteria, frequently dominant in hypersaline lakes (Liu et al. 2018) and adapted to nutrient‐poor conditions (Liu et al. 2018; Lin et al. 2017; Zhao et al. 2020) may therefore play a central ecological role across the lake's water column.

### 4.2 Alpha Diversity Patterns Along the Water Column

The alpha‐diversity patterns observed in Crater Lake contrast sharply with those reported from other soda lakes, underscoring the ecological variability of haloalkaline systems. In Crater Lake, diversity peaks in transitional oxic‐microoxic layers (5 and 20 m) and declines markedly toward the anoxic‐monimolimnion (23 m), where strong redox constraints appear to limit the number of taxa capable of persisting under chemically reduced conditions. This trend is opposite to that of Mono Lake, where diversity is lowest in the oxic‐mixolimnion dominated by Actinobacteria and increases substantially in the deep anoxic layers, where the accumulation of sulfide and ammonia supports expanded anaerobic metabolic niches (Humayoun et al. 2003).

Comparable but distinct diversity–environment relationships have been reported in other haloalkaline lakes. In the hypersaline Kulunda Steppe lakes, rising salinity (60–200 g/L) and high alkalinity (pH 10–10.3) greatly reduce taxonomic richness, selecting specialised halophilic and alkaliphilic lineages, mainly Proteobacteria and low‐G + C Gram‐positive bacteria, that maintain high functional activity despite reduced diversity (Foti et al. 2008). Similarly, Hamatai Lake exhibits a strong decline in Shannon diversity with increasing pH and salinity; alkalinity is the primary driver of community structure, favouring Firmicutes, Clostridia, and Gammaproteobacteria in high‐pH/high‐salinity zones (Liu et al. 2023). In contrast, Lonar Lake maintains consistently high bacterial diversity across the system, though sediments harbour significantly richer and more complex communities than the water column due to higher TOC/TN ratios and elevated concentrations of sulfate, ammonium, phosphate, and metals, which support metabolically diverse chemoheterotrophs (Paul et al. 2016).

Taken together, these comparisons show that Crater Lake follows a pattern most similar to alkalinity‐driven systems such as Hamatai and the Kulunda lakes, where physicochemical stress reduces diversity in extreme layers. However, unlike Mono Lake where anaerobic deep waters promote greater niche diversification, Crater Lake's deep anoxic environment appears more chemically restrictive. This highlights that haloalkaline stratification can generate opposing diversity gradients depending on lake‐specific combinations of redox chemistry, salinity, and organic matter availability.

### 4.3 Diversity, Structural Variation, and Distribution of PHB Depolymerases

The PHB depolymerases detected in the metagenomes and genomes of Crater Lake (Tables 4 and 5) span a broader taxonomic range than previously reported for haloalkaline systems. While most characterised depolymerases originate from members of Pseudomonadota, particularly the genus *Halomonas*, our findings reveal homologues affiliated with *Vreelandella*, *Thiomicrorhabdus*, and lineages within the phyla Chloroflexota and Candidatus Cloacimonadota, as well as the class Desulfobacteria (phylum Desulfobacterota). This expands the known diversity of PHB‐degrading lineages in extreme environments and aligns with recent evidence that uncultured taxa contribute substantially to PHA turnover in natural ecosystems (Viljakainen and Hug 2021).

*Halomonas* have been highlighted as cost‐effective hosts for producing bioplastics, biosurfactants, and industrial enzymes due to their tolerance to high salinity and alkaline pH (Ye and Chen 2021). Here, five PHB depolymerases were associated with *Vreelandella*, a member of the Halomonas_I phylogroup, suggesting that this genus may encode robust depolymerases capable of functioning under extreme conditions where other enzymes would denature. In *Halomonas titanicae* KHS3, Rodríguez et al. (2024) identified three PHB depolymerases, one of which (PhbZ1) possesses the same lipase box (GVSSG) found in the *Vreelandella*‐associated enzymes detected in this study. Those authors also reported a signal peptide for PhbZ1, consistent with the signal peptides identified in several *Vreelandella* depolymerases in our dataset.

Across all taxa, the PHB depolymerases identified here exhibit diverse structural features, including variation in lipase‐box motifs and the presence or absence of signal peptides that allow only tentative functional classification. In particular, differences in the X_1_ position of the lipase box (e.g., valine, phenylalanine, methionine) have been associated with distinctions among intracellular SCL, MCL, and extracellular depolymerases (Knoll et al. 2009; Jendrossek and Handrick 2002). While such patterns may indicate functional differentiation, these interpretations remain speculative and require experimental validation.

Taken together, the taxonomic breadth and structural variability observed among the PHB depolymerases suggest potential functional diversification across the lake's environmental gradients. However, sequence identity, lipase‐box composition, and signal‐peptide predictions alone are insufficient to confidently assign enzymatic types or ecological roles. To overcome these limitations and place the newly identified sequences within a broader evolutionary framework, we conducted a phylogenetic reconstruction (Section 4.3) that incorporated both the PHB depolymerase sequences identified in this study and a curated set of well‐characterised reference depolymerases. This approach enables more robust functional inference through clade‐level relationships.

### 4.4 Phylogeny of PHB Depolymerases

The phylogenetic analysis of the depolymerases identified in the metagenomes and genomes from the Crater Lake of Isla Isabel reveals a pattern of enzymatic diversification closely linked to the physicochemical gradients of the system. This lake exhibits persistent meromictic stratification, with high and stable temperatures (27°C–38°C), elevated salinity (0%–78%), alkaline pH (~10), and anoxic conditions in deeper layers, all of which promote metabolic and evolutionary differentiation of microbial communities (Emilio et al. 2022). These extreme conditions, together with the long‐term stability of the water column, create contrasting microhabitats that select for complementary metabolic strategies for the recycling of polymeric carbon along the oxic–anoxic gradient.

Depolymerases affiliated with *V. aquamarina*, abundant in surface samples (0–5 m), clustered with short‐chain‐length (SCL) extracellular enzymes from *P. lemoignei* , *D. acidovorans* , and R. pickettii (Jendrossek and Handrick 2002). The presence of a signal‐peptide and the conservation of the catalytic motif GVSSG indicate an extracellular localization and a likely role in the aerobic degradation of biopolymers in the oxygenated layer of the lake. The secretion of these enzymes represents an ecological advantage, as it enables the breakdown of complex polymers outside the cell, releasing soluble monomers that can be assimilated without compromising osmotic homeostasis (Zhang et al. 2022). In addition, halotolerant hydrolases often maintain catalytic stability under high salinity and alkaline pH, favouring their persistence and efficiency in extreme environments (Oren 2024). From a community perspective, the secretion of depolymerases into the extracellular environment may facilitate cooperative metabolic interactions among taxa, promoting efficient polymer remineralization and carbon recycling in the oxygenated zone of the lake (D'Souza et al. 2023). Such enzymatic specialisation is consistent with what has been observed in hypersaline ecosystems, where extracellular hydrolases contribute to maintaining biogeochemical balance under high radiation and osmotic stress (Oren 2024).

In contrast, the sequence Sed_PhbZV3, also affiliated with *Vreelandella*, lacks a signal‐peptide and lipase‐box, suggesting a potential relaxation of purifying selection or an ongoing process of functional diversification. This structural divergence may reflect an adaptive shift toward an intracellular or alternative role in polymer degradation under the reducing, anoxic sediment conditions, where selective pressure on extracellular enzymes is lower. Alternatively, the loss of these motifs may result from horizontal gene transfer or incomplete recombination events, which are frequent in haloalkaline environments with high genetic connectivity (Oren 2024; Cai et al. 2011). Together, these results suggest an evolutionary balance between functional conservation and adaptive plasticity, where *Vreelandella* depolymerases maintain a stable catalytic core under purifying selection, while variants such as Sed_PhbZV3 may represent divergent trajectories leading to specialisation or functional loss.

At intermediate depths of the water column (5–15 m), sequences affiliated with *T. indica*, a bacterium of the phylum Chloroflexota, and a bacterium of the order formed a well‐supported clade related to intracellular SCL depolymerases from *B. thuringiensis* . All sequences share the lipase‐box GSSLG and lack a signal‐peptide, supporting their role as intracellular enzymes involved in the recovery of endogenous carbon under microaerophilic or reducing conditions. Their depth distribution coincides with the oxygen–anoxia transition zone of the lake, where low oxygen availability and sulfate enrichment promote the dominance of chemolithotrophic and sulfur‐oxidising lineages that require flexible carbon utilisation strategies to thrive in an energetically constrained environment.

The sequences 00 m_PhbZW1 (*Wenzhouxiangella* sp.) and 05 m_PhbZP1 (*Pseudoalteromonas* sp.) clustered with medium‐chain‐length (MCL) intracellular depolymerases from *P. putida* and *P. oleovorans* . Both sequences contain the lipase‐box GFSQG and lack a signal‐peptide, suggesting intracellular activity associated with storage metabolism or internal polyester recycling. This pattern aligns with adaptations to hypersaline and oxidative surface waters, where extracellular secretion may be energetically inefficient due to osmotic pressure. In such microhabitats, retaining hydrolytic activity within the cell provides a physiological advantage for maintaining osmotic and energetic balance (Oren 2024).

Finally, the sequences 20 m_PhbZC1 (a bacterium of phylum Candidatus Cloacimonadota), Sed_PhbZCa2 (a bacterium of the phylum Chloroflexota), and 15 m_PhbZDf1 (a bacterium of the order Desulfobacterales), which possess the lipase‐box motifs GFSMG, GMSNG, and GASMG respectively, and signal peptides in the first two cases did not cluster with any reference depolymerases. This suggests the presence of divergent functional lineages that may represent evolutionary adaptations to deep, reducing environments where catalytic activity requires structural stability under high osmotic pressure and limited ionic hydration. Their distribution in anoxic, sulfidic layers indicates potential specialisation in polymer degradation under extreme energy limitation (Paul et al. 2016).

Altogether, these results indicate that the physicochemical structure of the lake, particularly its thermal stratification, redox gradient, and high alkalinity, act as an ecological and evolutionary driver promoting the functional diversification of bacterial depolymerases. Extracellular forms dominate in oxygenated zones, whereas intracellular forms prevail in anoxic environments, revealing a balance between conservation of a core catalytic architecture and ecological adaptation. This diversification pattern suggests that environmental pressures have shaped distinct enzymatic strategies while maintaining polymer degradation and carbon recycling as central processes in the haloalkaline ecosystem of Isla Isabel.

#### 4.4.1 Functional, Ecological and Biotechnological Implications

Beyond the phylogenetic patterns observed within each cluster, our results highlight a broader novelty in the diversity of PHB depolymerases in Crater Lake. Previous research on PHA and PHB metabolism in saline and haloalkaline systems has been strongly biassed toward cultivable representatives, particularly the genus *Halomonas*, which has been widely used as a model for both PHA production and biodegradation. However, emerging metagenomic evidence shows that PHB degradation in natural ecosystems involves phylogenetically diverse and often uncultured lineages that remain largely unexplored. In this context, our dataset expands the known taxonomic breadth of PHB depolymerases by identifying homologues in *Vreelandella*, *Thiomicrorhabdus*, the phyla Chloroflexota and Cloacimonadota, and the order Desulfobacterales, none of which had previously been linked to PHB degradation in haloalkaline lakes.

Moreover, the depth‐structured distribution of extracellular SCL depolymerases in oxic‐surface waters and intracellular SCL/MCL depolymerases in microoxic and anoxic layers indicates a functional partitioning that has not been reported in earlier studies. The combination of unique lipase‐box variants, presence or absence of signal‐peptides, and divergent phylogenetic placements suggests the existence of unrecognised enzymatic strategies adapted to the physicochemical gradients of Crater Lake.

Together, these findings advance beyond earlier work by demonstrating that PHB degradation in haloalkaline ecosystems is not restricted to a narrow set of well‐studied taxa but instead constitutes a distributed ecological function shaped by stratification, redox chemistry, and evolutionary diversification. While experimental validation is required to confirm the activity of these enzymes, the phylogenetic, structural, and ecological patterns uncovered here provide new hypotheses about polymer degradation and carbon recycling in soda lakes and highlight the importance of uncultured microbial lineages in these processes.

Building on this expanded phylogenetic and functional landscape, our results also provide a foundation for identifying candidates with potential biotechnological relevance. Although functional assignments are based on sequence inference alone, a limitation that requires future biochemical validation, the structural features identified here provide strong candidates for experimental characterisation. In particular, the extracellular SCL depolymerases affiliated with *V. aquamarina*, which retain conserved catalytic motifs and signal‐peptides and cluster with experimentally validated PHB‐degrading enzymes, emerge as strong candidates for heterologous expression and biochemical screening. Likewise, the divergent intracellular depolymerases from the phyla Chloroflexota and Cloacimonadota, as well as the order Desulfobacterales, with unique lipase‐box variants and depth‐specific distributions, represent promising targets for exploring novel substrate specificities or catalytic adaptations.

Future steps may include gene synthesis, expression in suitable bacterial hosts (e.g., *E. coli* , *Halomonas*, or halophilic chassis), and activity assays under high salinity and alkaline pH. Once the enzymatic activity of these depolymerases is validated, their biotechnological potential could be harnessed to close the life cycle of PHA (Ye and Chen 2021), linking production and degradation within a fully circular bioprocess (Zhou et al. 2023). While additional experimental characterisation remains necessary, the enzymes identified in this study provide a conceptual and functional framework for developing new extremophile‐based platforms for polymer depolymerization in industrial and environmental applications.

## 5 Conclusions

The metagenomic analysis of Crater Lake revealed a depth‐stratified microbial community, with surface waters dominated by Actinobacteriota and Pseudomonadota, and deeper layers enriched in Bacillota and Bacteroidota. These patterns reflect the lake's pronounced physicochemical gradients, particularly oxygen, salinity, and nutrient availability, and are consistent with observations from other haloalkaline soda lakes. Transitional depths exhibited higher diversity, highlighting the ecological importance of microhabitats at oxic‐anoxic interfaces.

PHB depolymerases identified in the metagenomes span a broader taxonomic range than previously reported, including Pseudomonadota (Halomonas, *Vreelandella*), *Thiomicrorhabdus*, Chloroflexota, Cloacimonadota, and Desulfobacterales. Their structural features, such as variation in lipase‐box motifs and presence or absence of signal‐peptides, indicate a functional partitioning along the water column, with extracellular depolymerases prevalent in oxic layers and intracellular forms in microoxic and anoxic zones. These patterns suggest both conservation of core catalytic motifs and adaptive diversification in response to environmental gradients.

Phylogenetic analyses further reveal potential evolutionary plasticity, with some depolymerases showing structural divergence that may reflect intracellular specialisation, relaxed selection, or adaptation to energy‐limited, reducing environments. Collectively, these findings indicate that PHB degradation in haloalkaline lakes is a distributed ecological function performed by phylogenetically diverse and often uncultured taxa, contributing substantially to polymer turnover and carbon recycling.

Finally, several extracellular SCL depolymerases, particularly those affiliated with *Vreelandella*, retain conserved catalytic motifs and signal‐peptides, representing promising candidates for heterologous expression and biotechnological applications under extreme conditions. Intracellular depolymerases from Chloroflexota, Cloacimonadota, and Desulfobacterales may offer additional opportunities to explore novel substrate specificities and catalytic adaptations. Overall, the depth‐structured diversity and functional specialisation of PHB depolymerases in Crater Lake provide a framework for understanding both ecological roles and potential industrial exploitation of these enzymes.

Despite these insights, functional assignments are based solely on sequence inference and phylogenetic placement, representing a key limitation. Experimental validation of enzymatic activity, substrate specificity, and environmental relevance remains necessary to confirm the predicted roles of these depolymerases. Future studies should focus on enzyme kinetics, structural modelling, and heterologous expression, particularly under high salinity and alkaline conditions, to explore both fundamental biochemical properties and industrial applicability.

The diversity and stability of the depolymerases identified here also provide a foundation for synthetic biology and metabolic engineering applications. Extracellular depolymerases from *Vreelandella* and other haloalkaliphiles represent promising candidates for incorporation into engineered microbial platforms for PHB degradation and recycling, potentially enabling circular bioprocesses for bioplastics. Additionally, the unique intracellular depolymerases from Chloroflexota, Cloacimonadota, and Desulfobacterales may serve as templates for protein engineering to develop enzymes with novel substrate specificities or enhanced catalytic performance. Collectively, these findings highlight both the ecological significance of PHB turnover in extreme environments and the translational potential of these enzymes for biotechnological innovation.

## Author Contributions

**Rina María González‐Cervantes:** conceptualization, writing – review and editing, supervision. **Humberto Garcia‐Arellano:** writing – review and editing, formal analysis, data curation, supervision. **Marcos López‐Pérez:** writing – review and editing, supervision, formal analysis. **Abigail Hernández‐Vázquez:** investigation, writing – original draft, methodology, formal analysis, conceptualization. **José Félix Aguirre‐Garrido:** conceptualization, funding acquisition, writing – review and editing, supervision, formal analysis. **Luis Mario Hernández Soto:** methodology, writing – review and editing. **José Abraham Canales Meza:** methodology, writing – review and editing.

## Ethics Statement

The authors have nothing to report.

## Consent

The authors have nothing to report.

## Conflicts of Interest

The authors declare no conflicts of interest.

## Data Availability

The raw metagenomic data were deposited under Bioproject accession number: PRJNA1240669. Genome sequences of *Vreelandella aquamarina*, strain VN105m have been deposited in GenBank under Bioproject accession number: PRJNA1389626. Genome sequences of *V. aquamarina*, strain HB105m have been deposited in GenBank under Bioproject accession number PRJNA1242893.
